# FAM83H-AS1 is a potential modulator of cancer driver genes across different tumors and a prognostic marker for ER/PR + BRCA patients

**DOI:** 10.1038/s41598-020-71062-2

**Published:** 2020-08-24

**Authors:** Magdalena Ríos-Romero, Alberto Cedro-Tanda, Mónica Peña-Luna, Marco Antonio Mancera-Rodríguez, Lizbett Hidalgo-Pérez, Mireya Cisneros-Villanueva, Fredy Omar Beltrán-Anaya, Rocío Arellano-Llamas, Silvia Jiménez-Morales, Luis Alberto Alfaro-Ruíz, Alberto Tenorio-Torres, Carlos Domínguez-Reyes, Felipe Villegas-Carlos, Elsa Ochoa-Mendoza, Alfredo Hidalgo-Miranda

**Affiliations:** 1 Laboratorio de Genómica del Cáncer, Instituto Nacional de Medicina Genómica, Periferico Sur 4809, Tlalpan, Arenal Tepepan, 14610 Ciudad de México (CDMX), Mexico; 2 Fundación de Cáncer de Mama, FUCAM, Ciudad de México, Mexico; 3 Programa de Doctorado de Ciencias Biológicas, Universidad Nacional Autónoma de México, Ciudad de México, Mexico

**Keywords:** Breast cancer, Oncogenes

## Abstract

Breast cancer (BRCA) is a serious public health problem, as it is the most frequent malignant tumor in women worldwide. BRCA is a molecularly heterogenic disease, particularly at gene expression (mRNAs) level. Recent evidence shows that coding RNAs represent only 34% of the total transcriptome in a human cell. The rest of the 66% of RNAs are non-coding, so we might be missing relevant biological, clinical or regulatory information. In this report, we identified nine novel tumor types from TCGA with FAM83H-AS1 deregulation. We used survival analysis to demonstrate that FAM83H-AS1 expression is a marker for poor survival in IHC-detected ER and PR positive BRCA patients and found a significant correlation between FAM83H-AS1 overexpression and tamoxifen resistance. Estrogen and Progesterone receptor expression levels interact with FAM83H-AS1 to potentiate its effect in OS prediction. FAM83H-AS1 silencing impairs two important breast cancer related pathways: cell migration and cell death. Among the most relevant potential FAM83H-AS1 gene targets, we found p63 and claudin 1 (*CLDN1*) to be deregulated after FAM83H-AS1 knockdown. Using correlation analysis, we show that FAM83H-AS1 can regulate a plethora of cancer-related genes across multiple tumor types, including BRCA. This evidence suggests that FAM83H-AS1 is a master regulator in different cancer types, and BRCA in particular.

## Introduction

Breast cancer (BRCA) is a serious public health problem, as it is the most frequent malignant tumor in women worldwide. According to GLOBOCAN^1^, at least 1.67 million new cases and a total of 522,000 deaths are reported globally.

BRCA is a phenotypically heterogenic disease; with well-defined histological types and protein markers, such as Estrogen receptor (ER), Progesterone Receptor (PR) and membrane receptor HER-2. The physical and phenotypical BRCA heterogeneity is also reflected at the molecular, particularly at gene expression (mRNAs) level. This heterogeneity has been extensively studied, and evidence shows that breast cancer comprises four intrinsic groups: Luminal A, Luminal B, HER-2 enriched and Basal-like tumors^2,3^.

Molecular classification has been an important milestone in BRCA biology, as it has been used to differentiate aggressive and non-aggressive tumors, metastatic potential, clinical prognosis and survival, among other relevant cancer-related features^4^. Additionally, therapy response-associated expression profiles are now available. This expression profiles are able to predict if a patient can benefit from chemotherapy or anti-hormonal therapy^5^. These useful clinical advances are focused on coding RNAs profiles only; however, recent evidence show that coding (messenger) RNAs represent only 34% of the total transcriptome in a human cell^6^. The rest of the 66% of RNAs are non-coding, so we might be missing relevant biological, clinical or regulatory information if we only focus on messenger RNA.

In this regard, recent papers have focused on the role of long non coding RNAs (lncRNAs) in cancer biology^7–10^ and in the role of specific lncRNAs in breast cancer.

FAM83H-AS1 is a lncRNA whose expression impairs important cancer-related pathways such as cell proliferation, migration, invasion and cell death in lung, colorectal, glial, bladder, ovarian and cervical cancer cells^11–16^. At the molecular level, one report showed that MET/EGFR signaling is regulated by FAM83H-AS1, and^16^ showed that FAM83H‐AS1 epigenetically silenced *CDKN1A* by binding to EZH2 in glioma cells.

In addition, two reports showed that FAM83H antisense RNA 1 (FAM83H-AS1) is deregulated BRCA samples. High expression of FAM83H-AS1 indicated an unfavorable prognosis in luminal type BRCA^12^ and early-stage BRCA^16^. Altogether, this evidence shows that FAM83H-AS1 is an important actor in cancer biology. In this paper, we identified nine novel tumor types from TCGA with FAM83H-AS deregulation, and used a multivariate Cox regression analysis to demonstrate that FAM83H-AS1 expression is a marker for poor survival in Progesterone receptor (PR) positive BRCA. We found a significant correlation between FAM83H-AS1 overexpression and tamoxifen resistance in luminal BRCA patients. Using Kaplan–Meier and Cox regression analysis, we found that estrogen and progesterone receptor expression levels interact with FAM83H-AS1 to potentiate its effect in OS prediction. Using FAM83H-AS1 short hairpin knockdown coupled with microarray analysis, we demonstrate that FAM83H-AS1 silencing impairs two important breast cancer related pathways: cell migration and cell death. We further validate this phenotypic effect with in vitro migration and caspase 3 assays. Among the most relevant potential FAM83H-AS1 gene targets, we found p63 and claudin 1 (*CLDN1*) to be deregulated after FAM83H-AS1 knockdown. Using correlation analysis, we show that FAM83H-AS1 can regulate a plethora of cancer-related genes across multiple tumor types, including BRCA.

## Results

### FAM83H-AS1 is deregulated in multiple tumor types

**Figure 1:**
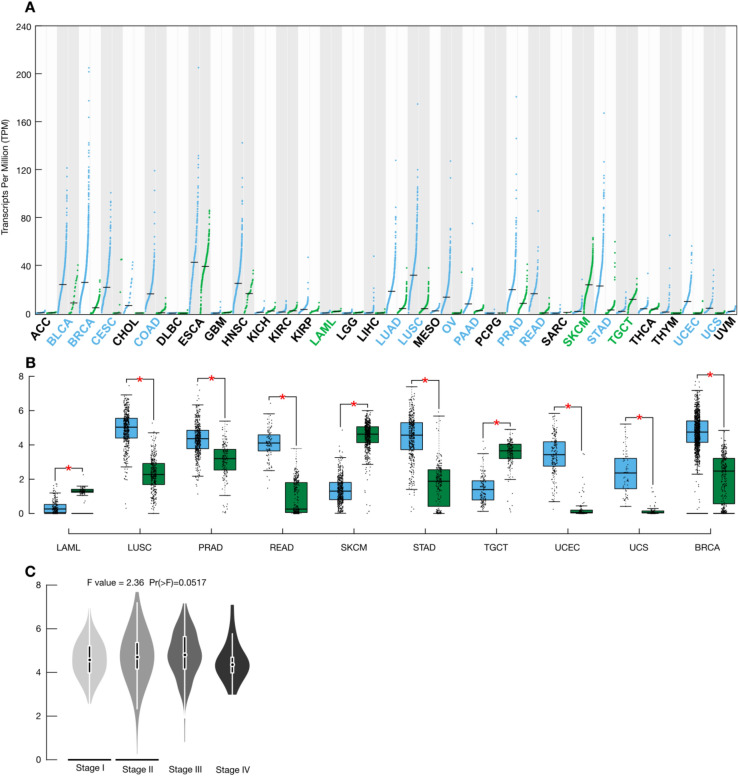
FAM83H-AS1 expression is altered in multiple human tumors. (**A**) FAM83H-AS1 expression levels (TPM) in 33 tumors from the TCGA database. In green are shown normal tissue samples, in blue tumor samples. (**B**) FAM83H-AS1 is aberrantly expressed in nine not previously reported tumors. Color code as in A. (**C**) FAM83H-AS1 is enriched in locally advanced BRCA clinical stages (II and III).

Multiple studies have related FAM83H-AS1 high expression levels with different tumors, including luminal breast cancer^11–16^. These findings suggest an important role for FAM83H-AS1 in cancer tumor biology. We therefore screened FAM83H-AS1 expression levels in the TCGA database, which comprises data from 33 different tumor types and the correspondent normal tissues. As expected, we found significant FAM83H-AS1 expression deregulation in 16 different tumor types (Fig. 1A) (Log2FC > 1; *p* < 0.01). Some of these FAM83H-AS1 expression deregulation data has been reported previously^11,12,16^, but we have also found significant deregulation of FAM83H-AS1 in nine additional tumor types (Fig. 1B) (Log2FC > 1; *p* < 0.01). Interestingly, FAM83H-AS1 was up-regulated in 15 different tumor types, but down-regulated in acute myeloid leukemia (LAML), suggesting a different mechanism for this particular malignancy (Fig. 1B).

### FAM83H-AS1 expression level is enriched in BRCA locally-advanced tumors

It was reported that FAM83H-AS1 expression is a prognostic marker for luminal breast cancers^12^. We were interested to see if FAM83H-AS1 expression was more widely associated with BRCA tumors, since we and others found alterations for this lncRNA in a large number of malignancies. As shown in Fig. 1B, FAM83H-AS1 is significantly up-regulated in all BRCA patients, not only in the luminal subtype BRCA. FAM83H-AS1 over-expression is also marginally associated with BRCA locally advanced (II and III) clinical stages (one-way ANOVA; *p* = 0.05) (Fig. 1C).

### FAM83H-AS1 is a prognostic marker for ER and PR positive BRCA and its expression is related with tamoxifen resistance

**Figure 2:**
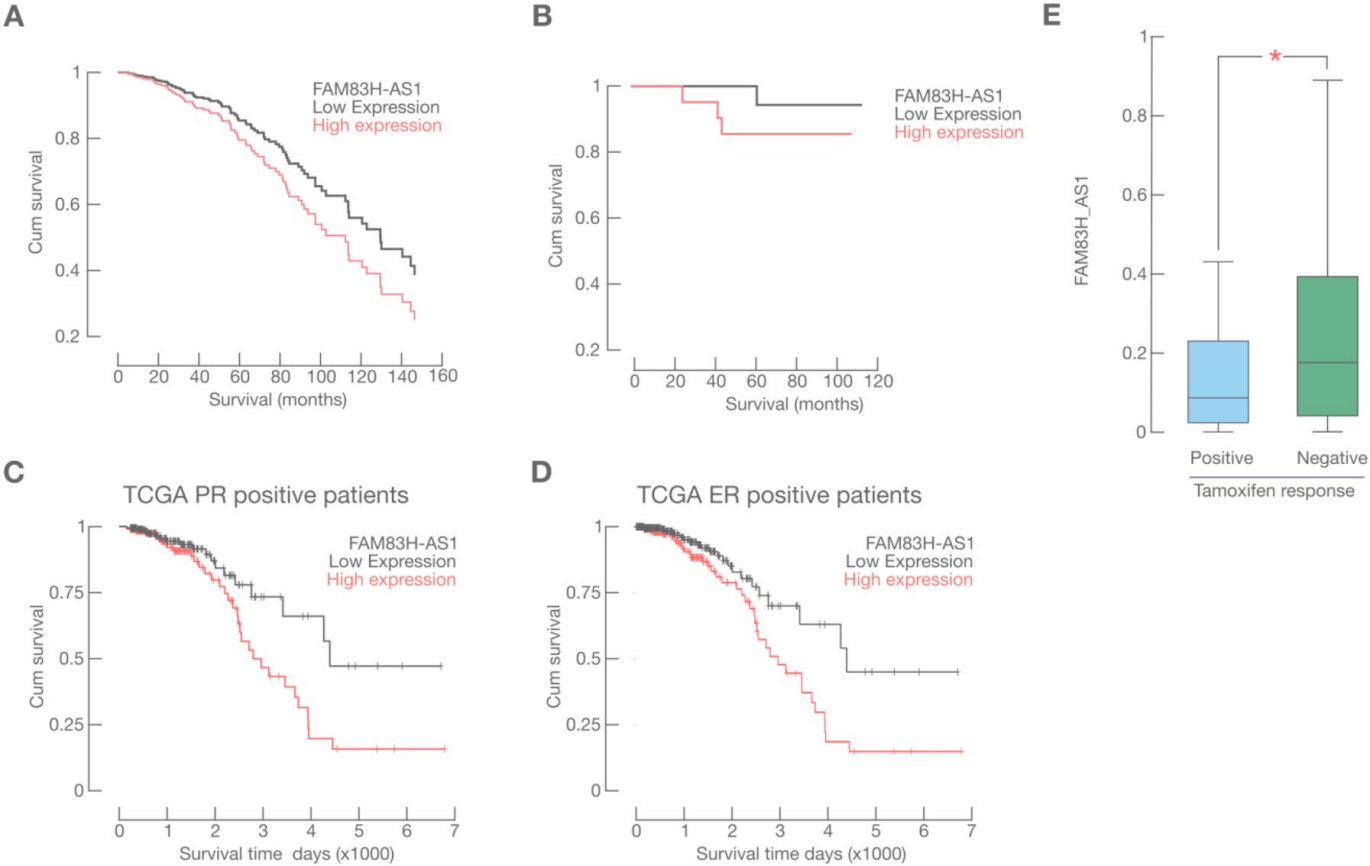
FAM83H-AS1 over-expression is a marker for poor prognosis in ER/PR BRCA patients. (**A**) Overall survival analysis (Cox Regression) for the BRCA TCGA cohort. (**B**) Kaplan–Meier analysis for our independent BRCA cohort. (**C**) Kaplan–Meier analysis for PR and **D**, ER positive BRCA patients from the TCGA cohort. (**E**) FAM83H-AS1 expression is associated with tamoxifen resistance in BRCA patients.

Altogether, these widespread alterations in FAM83H-AS1 expression suggested that its expression could be a prognostic biomarker for all BRCA subtypes: but as mentioned above, FAM83H-AS1 was previously reported to have particular prognostic association with the BRCA luminal subtype^12,16^. To test if FAM83H-AS1 is a widespread or a luminal specific prognostic marker in BRCA, we first screened FAM83H-AS1 expression as a prognostic marker for all BRCA tumors. We did not find significant association with poor OS in the Cox regression model (n = 743; 95% CI [0.442–1.15] Cox *p* value = 0.09); however, we observed a clear tendency in poor survival prognosis in the FAM83H-AS1 high expression group (see Fig. 2A). We further validated these results in an independent Mexican patient cohort (Fig. 2B) with all the BRCA subtypes. The general clinical features of this cohort are listed in Table 1.

**Table 1:** Clinical-pathological characteristics of population (n = 42).

Characteristic	Class	Frequency	Percent
Age	0–50	19	45.2
	51–100	23	54.8
Tumor grade	I	3	7.1
	II	26	61.9
	III	9	21.4
	SV	4	9.5
Clin. stage	0, IA, IIA, IIB	37	88.1
	IIIA, IIIB, IIIC	5	11.9
ER	Positive	25	59.5
	Negative	17	40.5
PR	Positive	23	54.8
	Negative	19	45.2
HER2	Positive	13	31
	Negative	29	69
Lymph Nodes	Positive	18	42.9
	Negative	10	23.8
	NA	14	33.3
Recurrence	Positive	7	16.7
	Negative	35	83.3
Metastasis	Positive	7	16.7
	Negative	35	83.3

In this Mexican cohort, none of these clinical variables were significantly correlated with FAM83H-AS1 expression level.

We then tested if this effect was due to FAM83H-AS1 interacting with other significant clinical and survival-related variables, in particular, luminal type-related. FAM83H-AS1 predictive value was significant when interacting with Immunohistochemistry (IHC)-detected Progesterone receptor (n = 743; HR = 1.55; 95% CI [1.005–2.376] Cox *p* value = 0.047) (supplementary Table 1). Marginal, but not significant, association was observed with ER status (supplementary Table 2). Kaplan–Meier analysis of PR (logrank; *p* = 0.014) or ER positive patients (logrank; *p* = 0.006) showed significant association poor OS when FAM83H-AS1 was over-expressed (Fig. 2C, D). No effect in survival rate was seen when FAM83H-AS1 was over-expressed in PR and ER negative patients (supplementary Figs. 1 and 2).

**Table 2:** Examples of differentially expressed genes in the FAM83H-AS1 high versus. low BRCA sample.

Description	Pathway	Fold change (log2)	*p*adj-value
Myosin Light chain 2	Tight junction signaling	4.268825581	3.38E−52
Myosin heavy chain 2	WNT signaling pathway	3.267559072	9.51E−42
Actin alpha 1	WNT signaling pathway	2.247491528	1.06E−30
Leptin	Cell migration	− 2.03868572	1.10E−08
MicroRNA 181	Cell migration	− 1.757287497	8.07E−06
Nuclear receptor 1 H4	Cell migration	− 1.923864676	1.47E−05
Fibroblast Growth Factor 4	MAPK signaling/Focal adhesion	− 1.60587355	9.78E−08
Fibroblast Growth Factor 21	MAPK signaling/Focal adhesion	− 1.813231804	3.74E−05
Claudin 17	Epithelial to mesenchymal transition	− 1.609953043	0.00048284
Insulin like growth factor binding protein 1	PI3K pathway	− 3.010911164	1.75E−11
Cadherin 9	Cadherin signaling	− 1.579133916	2.44E−05
Hydroxy-delta-5-steroid dehydrogenase	Steroid synthesis	− 1.961005522	1.57E−10
UDP glucuronosyltransferase family 1 member A1	Steroid synthesis	− 1.554304254	0.001164245
Alcohol dehydrogenase 1A (class I) alpha polypeptide	Drug metabolism	− 2.556929166	4.05E−09
Cytochrome P450 family 2 subfamily A member 6	Drug metabolism	− 2.989324666	3.13E−09
Deathassociated protein kinase 3	Cell death regulation	− 0.277	1.15E−04
Tumor protein p53	Cell death regulation	0.139	5.00E−02
TNF receptor superfamily member 11b	Cell death regulation	− 0.126	4.22E − 01
BCL2 associated X, apoptosis regulator	Apoptosis regulation	− 0.136	6.64E−02
BH3-like motif containing cell death inducer	Cell death regulation	− 2.779	1.07E−11
Phosphate and tensin homolog	Cell survival signaling	− 0.19	1.46E−03

This data strongly suggest that FAM83H-AS1 is an independent prognostic marker for OS in PR positive BRCA subtype and confirms with further statistical analyses, previous findings made by^12^.

We found a significant association with IHC-detected PR and ER status in the survival context (Fig. 2 C, D; supplementary Table 1). We then analyzed tamoxifen treatment resistance or sensitivity in our independent cohort (Fig. 2E), and we found that FAM83H-AS1 overexpression was significantly related with poor tamoxifen initial response (n = 42; OR = 3.9; one-tailed F-exact test; *p* = 0.045).

We did not find a significant prognosis association for 8 unreported tumors shown above in the Gene expression Profiling Interactive Analysis (GPIA) database (see methods). We could determine, however, a strong correlation between high FAM83H-AS1 expression and poor OS in skin cutaneous melanoma (SKCM) patients (n = 461; HR = 1.6; log-rank test, *p* = 0.0003) (Supplementary Fig. 3).

**Figure 3:**
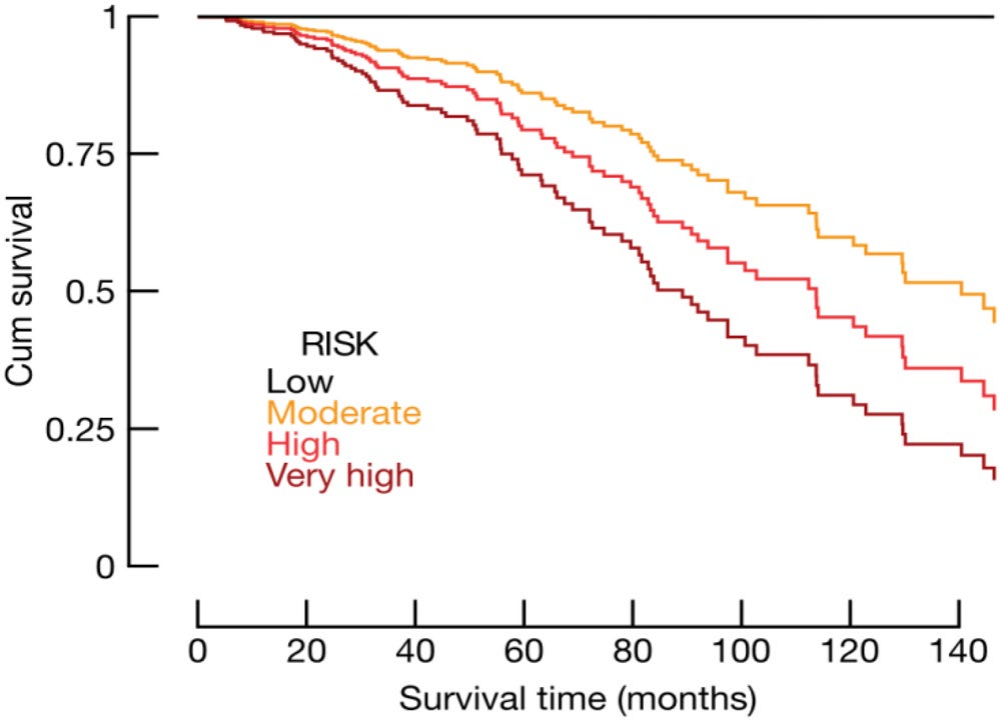
ER/PR expression potentiates FAM83H-AS1 death risk prediction. Expression levels from ER and PR were calculated using Allread scores. FAM83H-AS1 expression levels were calculated from the quartile distribution of the log2 values (TANRIC). We then multiplied Allread scores (17) and FAM83H-AS1 expression levels in order to define risk groups (see methods and main text).

### ER and PR expression levels potentiate FAM83H-AS1 prediction of survival in BRCA patients

In order to further characterize our previous finding regarding ER and PR status, and its association with FAM83H-AS1 in BRCA prognosis, we built a risk model taking into account ER, PR and FAM83H-AS1 expression levels in the same analysis. This model fundamentally displays ER, PR and FAM83H-AS1 interaction and potentiation of the poor OS prediction in BRCA.

We first calculated the Allred score^17^ to identify IHC ER and PR level of positivity*, ie.*ER and PR expression levels from TCGA data. We then obtained FAM83H-AS1 expression levels from the TCGA cohort and divided it in four strata (quartiles). We then multiplied these two values (Allred score values and FAM83H-AS1 quartile values), and the product was a new risk score. We obtained four risk groups with the above described method, shown in Fig. 3. As shown here, combination of very high FAM83H-AS1 and ER/PR expression levels potentiates high risk of decease in the TCGA cohort (Kaplan–Meier model, one tailed logrank; *p* = 0.034). Cox hazard proportional risks for decease in these four groups were: 0 for low risk, 25 for moderate risk, 39 for high and 57 for very high risk. This data confirms a strong interaction between ER, PR and FAM83H-AS1 expression levels in BRCA. Interaction between these three variables potentiates poor OS prediction in BRCA patients, and possibly other hormone-related human tumors.

### FAM83H-AS1 potentially regulates a plethora of cancer-related genes

**Figure 4:**
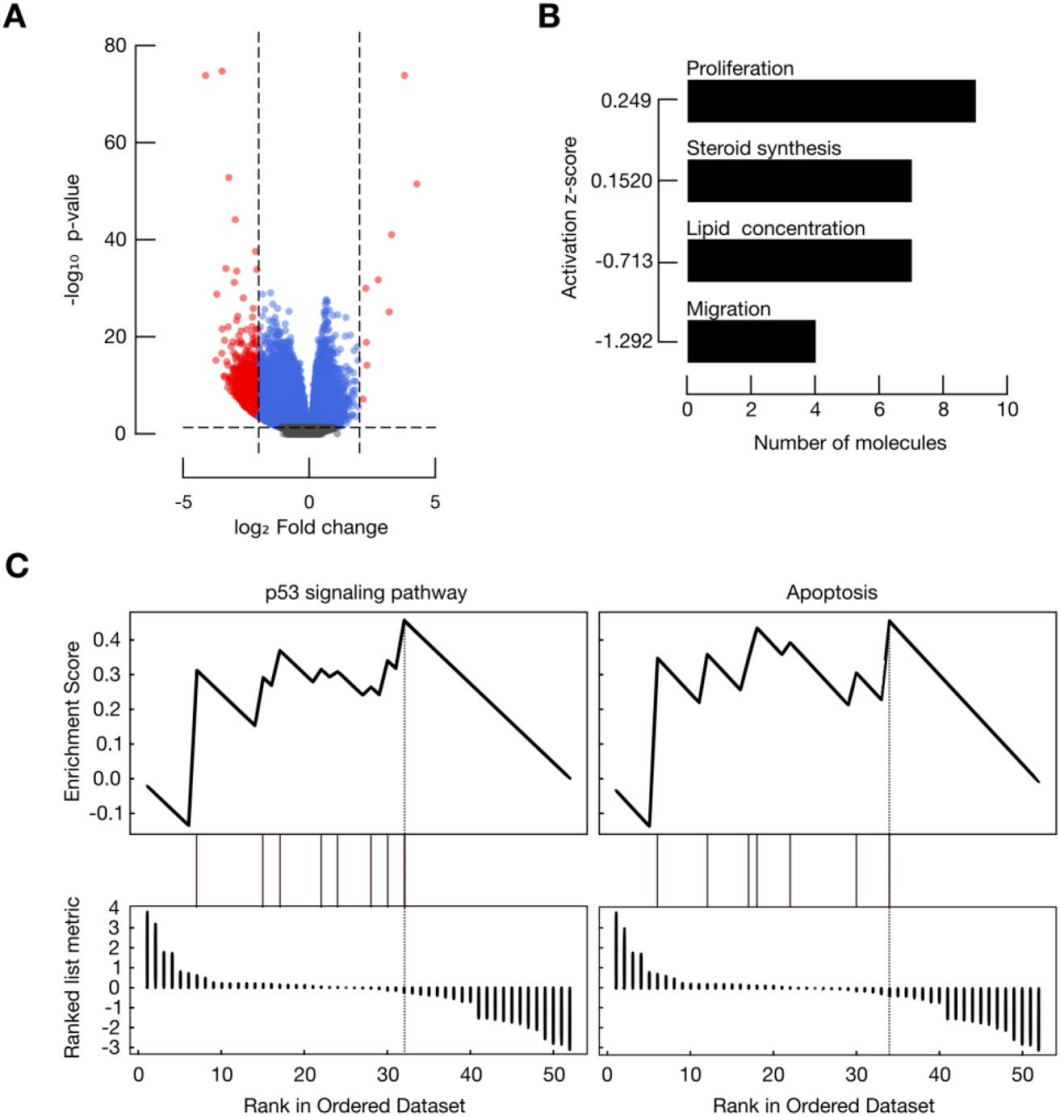
High FAM83H-AS1 levels in BRCA samples are correlated with down-regulation of cancer-related inhibitors. (**A**) Volcano plot depicting differentially expressed transcripts in high versus low FAM83H-AS1 levels. Points in grey = non-significant; blue = *p* value significant; red = fold change, and *p* value significant. (**B**) Migration-related and steroid metabolism genes are significantly down-regulated when FAM83H-AS1 is highly expressed in BRCA. (**C**) GSEA analysis showed enrichment for apoptosis cell death and p53-signalling pathways in the FAM83H-AS1-high samples.

In order to better understand FAM83H-AS1 role in BRCA, we performed a differential expression analysis using the TCGA BRCA cohort. We compared high versus low FAM83H-AS1 RNA expression level samples (see methods). We found 2,668 differentially expressed genes between these two groups (Log2FC > 1.5 and −  <1.5; *p*.adj-value < 0.01). The vast majority of these transcripts (98.6%; 2,631 RNAs) were found to be down-regulated in this analysis. These results might suggest candidate target genes for FAM83H-AS1 (Fig. 4A).

Among the down-regulated RNAs, we identified several cancer-related transcripts, such as: fibroblast growth factor 4 (*FGF4*); fibroblast growth factor 21 (*FGF21*); leptin (*LEP*), Claudin 17 (*CLDN17*); cadherin 9 (*CDH9*) Tumor Necrosis Factor receptor (*TNFRSF11B*); BCL2-associated X (*BAX*); Tumor protein p53 (*TP53*) and Phosphatase and tensin homolog (*PTEN*) (see Table 2).

After pathway enrichment analysis, we found a significant down-regulation of cellular migration, synthesis of steroids and lipid metabolism (Fig. 4B). Gene set enrichment analysis (GSEA) also showed alterations in apoptosis and p53-signalling pathways (Fig. 4C). Taken together, this evidence suggests an important regulatory role for FAM83H-AS1 in cancer-related pathways.

### FAM83H-AS1 is present both in nucleus and cytoplasm in ER/PR BRCA cells

**Figure 5:**
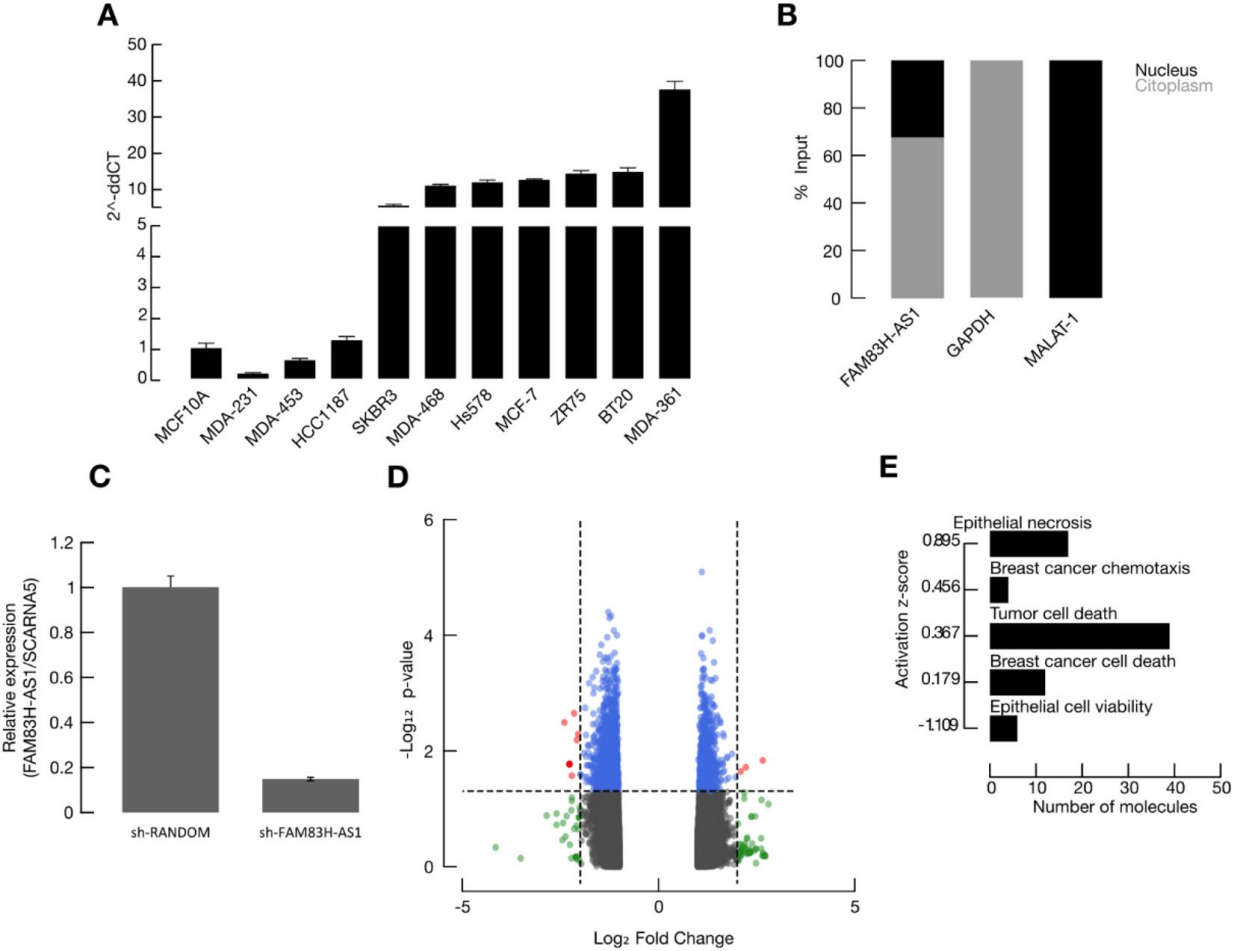
FAM83H-AS1 knockdown in MCF7 cells is associated with de-regulation of multiple cancer-related genes. (**A**) FAM83H-AS1 expression profile in breast cancer cell lines. (**B**) FAM83H-AS1 is localized both in nucleus and cytoplasm, but is enriched in cytoplasm in MCF7 cells. (**C**) short hairpin RNA silencing of FAM83H-AS1 in MCF7 cells. (**D**) sh-mediated silencing of FAM83H-AS1 induces differential expression in 415 genes in MCF7 cells. Grey = non-significant; blue = *p* value significant; green = fold change significant; red = *p* value, and fold change significant. **E,** Cellular migration and cell death are two significantly enriched pathways after FAM83H-AS1 silencing in MCF7 cells.

To further characterize FAM83H-AS1 functional role in BRCA, we measured its expression in nine breast cancer cell lines, including: MDA-MB-231, 468, 453, HCC1187, MCF7, SKBR3, BT20, Hs578, ZR75 and one non-transformed cell line, MCF10 (Fig. 5A). As expected, FAM83H-AS1 was up-regulated in transformed cell lines. We then performed cellular fractionation assays in MCF7 cells and detected its enrichment in the cytoplasmic fraction (67.3% of total input RNA) (Fig. 5B). LncATLAS screening further confirmed this observation, as MCF7 cells display both FAM83H-AS1 cytoplasmic and nuclear localization (Supplementary Fig. 4).

### FAM83H-AS1 knockdown deregulates 415 transcripts expression in MCF7 cells

In order to gain further insight into the potential FAM83H-AS1 targets, we performed sh-mediated FAM83H-AS1 silencing experiments in MCF7 cells. As shown in Fig. 5C, we obtained 85% of silencing efficiency after 48 h of plasmid transfection. After knockdown, we performed microarray experiments in MCF7 cells. We identified 415 differentially-expressed genes in the FAM83H-AS1-silenced cells (FC > 1.5 and −  <1.5; *p* value < 0.05) (Fig. 5D). Key cancer-related transcripts were identified, such as *CLDN1, TP63, FGF14, DDX60* and *DRAM* (see Table 3). 262 transcripts were found to be down-regulated whereas 153 were up-regulated (Fig. 5D; Table 3). Among the most up-regulated RNAs, we found *TP63* and *CLDN1.* These genes can also be potential candidate target genes for FAM83H-AS1 activity.

**Table 3:** Examples of differentially expressed genes after FAM83H-AS1 knockdown in MCF7 cells.

Gene symbol	Description	Pathway	Fold change (sh-FAM83H-AS1 vs. sh-RANDOM)	*p* value
CLDN1	Claudin 1	Epithelial to mesenchymal transition	1.64	0.023413
TP63	Tumor protein p63	Apoptosis signaling	1.56	0.017146
H3F3C	H3 histone, family 3C	DNA replication	1.51	0.033872
FGF18	Fibroblast growth factor 18	MAPK signaling/Focal adhesion	− 1.43	0.000613
DDX60	DEAD (Asp–Glu–Ala–Asp) box polypeptide 60	Cell death regulation	− 1.45	0.008851
IFI6	Interferon, alpha-inducible protein 6	Interferon signaling	− 2.4	0.003207
DRAM1	DNA-damage regulated autophagy modulator 1	Cell death regulation	− 1.54	0.018695
GPER1	G-protein coupled estrogen receptor 1	Endocrine resistance	− 1.31	0.0286
PARP9	Poly(ADP-ribose) polymerase family member 9	DNA repair	− 1.34	0.001424
GSTM3	Glutathione S + S transferase mu 3 (brain)	Drug metabolism	− 1.49	0.012426

### FAM83H-AS1 silencing impairs cellular migration and apoptosis

Pathway enrichment analysis showed that the most activated cellular processes in the sh-FAM83H-AS1 condition are: migration and cellular motility (29 altered molecules; *p* value range < 0.01) and cellular death pathways (49 molecules; *p* value range < 0.01) (Fig. 5E). These data are in accordance with our previous differential expression results in BRCA samples (Fig. 4), further supporting a master regulatory role for FAM83H-AS1 in breast cancer cells.

**Figure 6:**
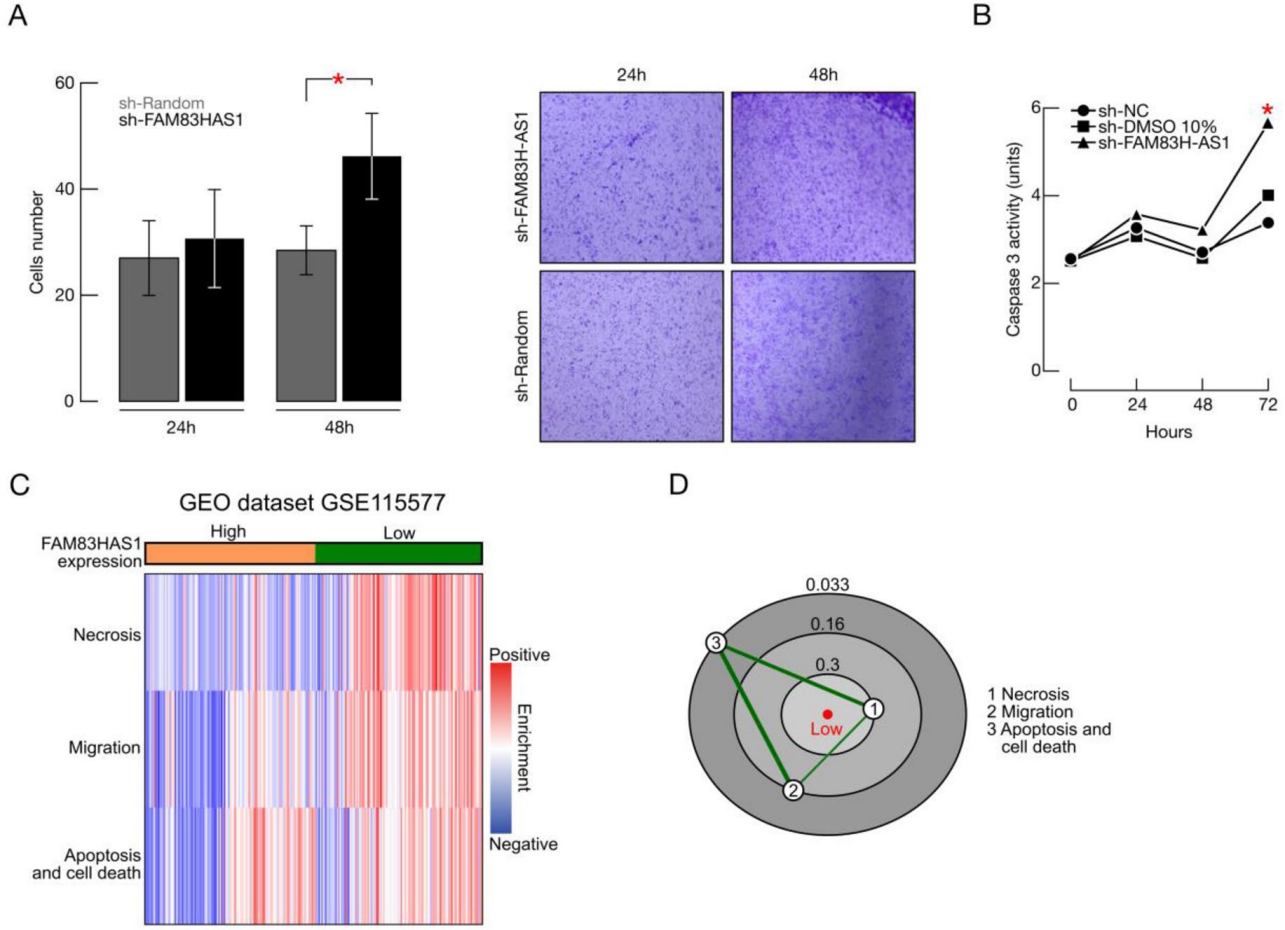
FAM83H-AS1 knockdown impairs cellular migration and induces cell death in breast cancer cells. (**A**) Transwell in vitro migration assays in MCF7 cells. (**B**) Caspase 3 activity assays in MCF7 cells. (**C**) Heatmap of the 3 gene sets enriched in the high FAM83H-AS1 expression group (blue) compared with that of FAM83H-AS1 low expression samples (green). (**D**) Constellation Map of the 3 gene sets. Three connected clusters of gene sets (migration, apoptosis and cell death and necrosis pathways) are detected in the low-FAM83H-AS1 group.

We then aimed to functionally validate that both cellular processes are altered after FAM83H-AS1 silencing. We thus performed Transwell migration assays and caspase 3 activity assays in MCF7 cells. As shown in Fig. 6A, MCF7 cells migration significantly increases after sh-FAM83H-AS1 transfection (one tailed T-test; *p* = 0.035). We then performed matrigel-invasion assays and observed an increase 24 h after transfection, but this observation did not reach statistical significance. (Supplementary Fig. 5). Furthermore, we did not find significantly altered invasion related genes or pathways in the microarray assays nor the differential expression analysis in the TCGA cohort, further suggesting that FAM83H-AS1 is not involved in invasion in this model.

Caspase 3 assays identified a significantly increase in enzymatic activity (T-test; *p* = 0.032) after 72 h of FAM83H-AS1 silencing, which corroborates its role in apoptosis mediated cell death. Caspase 3 is the primary activator of apoptotic DNA fragmentation^18^. Significant increase in caspase 3 activity in the sh-FAM83H-AS1 condition suggests FAM83H-AS1 regulation of late stage apoptosis (Fig. 6B).

### Migration and cell death alterations are enriched in the FAM83H-AS1 low expression group in BRCA samples

To further validate our previous in vitro results, we performed single sample GSEA (ssGSEA) in a BRCA independent cohort (Gene Expression Omnibus dataset GSE115577) (see methods). In this approach, gene sets are ranked according to absolute expression values in every sample, rather than by a comparison with another sample. We first stratified the GSE115577 cohort onto two groups: samples with high levels of FAM83H-AS1 RNA, and samples with low FAM83H-AS1 levels, using the quartile approach described above. We then aimed to know if the gene sets corresponding to migration, apoptosis and other cell death processes, like necrosis, were significantly enriched in any of these two FAM83H-AS1 expression groups. As expected, we found a significant enrichment of the migration (Normalized Mutual Index [NMI] score = 0.16; AUC = 0.79; *p* value = 9.18e−15) and apoptosis (NMI score = 0.033; AUC = 0.61; *p* value = 0.001) processes in the FAM83H-AS1-low expression group (Fig. 6 B). Interestingly, we also found a strong enrichment of the necrosis pathway in this BRCA cohort (NMI score = 0.3; AUC = 0.83; *p* value = 1.3e−18).

### FAM83H-AS1 and its potential target genes are co-deregulated across multiple tumor types

**Figure 7:**
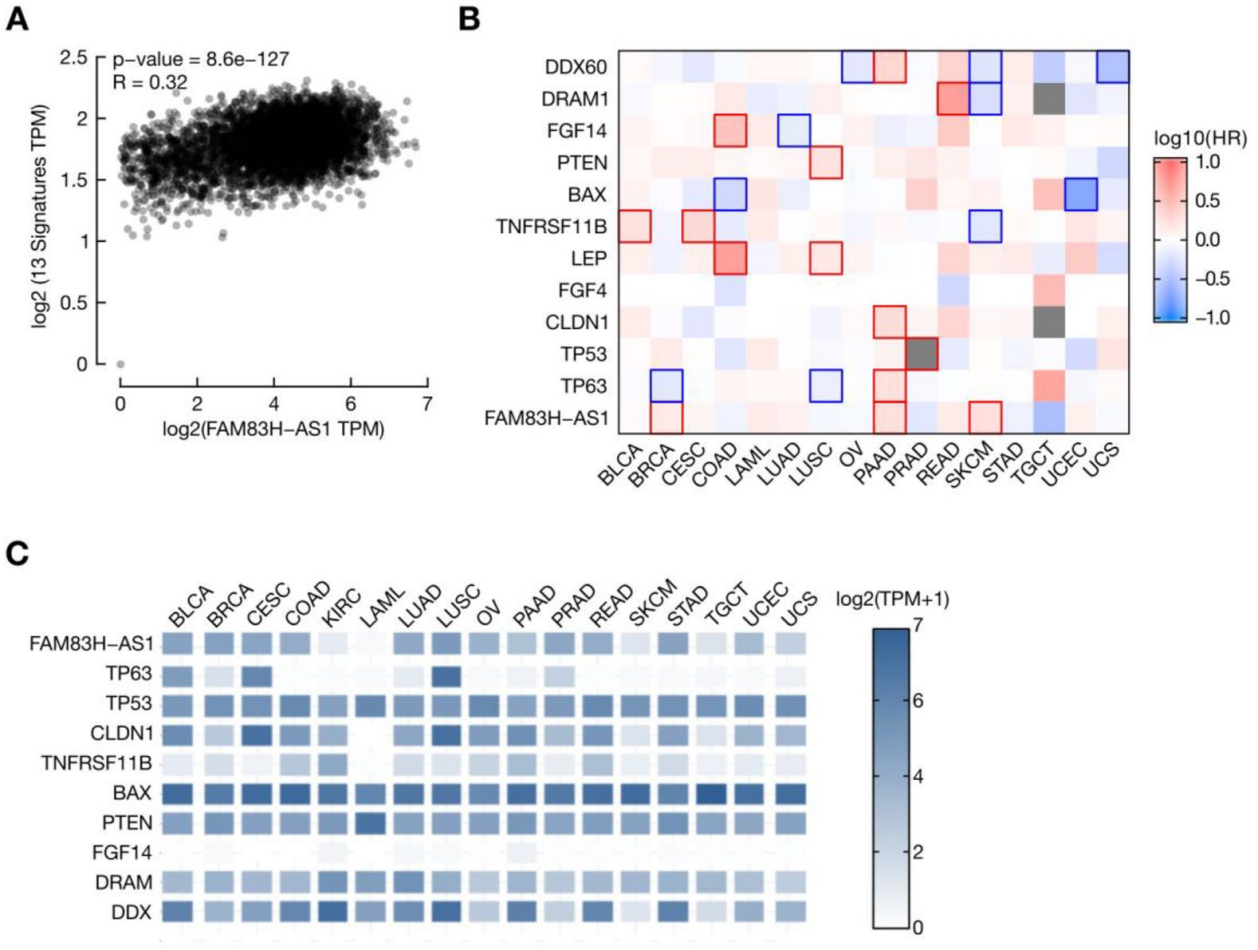
The expression of master regulators of cancer, such as p53 and p63, are dependent of FAM83H-AS1. (**A**) FAM83H-AS1 expression levels are strongly correlated with 12 potential target genes (TP53, TP63, BAX, CLDN1, CLDN17, CDH9, TNFRSF11B, PTEN, FGF4, FGF14, DRAM, DDX60 ) across 13 different tumors ( BLCA, BRCA, CESC, COAD, LUAD, LUSC, PAAD, PRAD, READ, OV, STAD, UCEC, UCS, SKCM). (**B**) Overall survival heatmap, depicting that over-expression of FAM83H-AS1 confers high risk of death in BRCA, PAAD and SKCM patients. (**C**) FAM83H-AS1 and its potential target genes are deregulated in 17 different tumors.

We found that FAM83H-AS1 is up-regulated not only in BRCA, but also in other tumors (see Fig. 1). We reasoned that, if up-regulated, FAM83H-AS1 may be exerting similar regulatory roles in other tumors as well. In order to show this, we correlated FAM83H-AS1 expression levels with its potential target genes (BAX, CLDN1, CLDN17, DRAM, DDX60, FGF4, FGF14, LEP, PTEN, TNFRSF11B, TP53 and TP63). As shown in Fig. 7A, FAM83H-AS1 is strongly correlated with these coding genes across multiple tumor types, namely BLCA, BRCA, CESC, COAD, LAML, LUAD, LUSC, OV, PAAD, PRAD, READ, SKCM, STAD, TGCT, UCEC and UCS. We then calculated the hazard ratio (HR) of decease event related to FAM83H-AS1 and its potential targets expression in 16 different tumor types from TCGA (Fig. 7B). We found a significant association (log-rank test; *p* < 0.05) of FAM83H-AS1 overexpression and a high decease HR in BRCA, PAAD and SKCM (Fig. 7B). The expression of FAM83H-AS1 potential targets is also associated with high or low HR in these tumors. Some of the coding genes that we either found in the differential expression analysis (Fig. 4) or after FAM83H-AS1 knockdown in MCF7 cells (Fig. 5D) are also co-deregulated in other tumor types (Fig. 7C). Altogether, this evidence suggests a wide master regulatory role for FAM83H-AS1 not only in ER/ PRBRCA, but in other tumor types, such as PAAD and SKCM.

## Discussion

Long non-coding RNAs are molecules that exert numerous roles in human cancers, as their biological activities involve regulation of cell proliferation, cell death, differentiation, migration and invasion. Deregulation in lncRNAs expression has also been associated with clinical outcome. LncRNAs can affect expression of thousands of genes, so they are regarded as key master regulators^7–10^.

In this work, our aim was to investigate a wider de-regulation for FAM83H-AS1 expression in tumors, focusing on its functional and clinical role in breast cancer and the identification of potential FAM83H-AS1 targets. We found that FAM83H-AS1 was overexpressed in nine different tumor types in the TCGA database. In particular, FAM83H-AS1 is overexpressed and significantly correlated with a worse clinical outcome in PR positive (detected by immunohistochemistry) BRCA subtypes, in the TCGA breast cancer cohort. One previous report^12^ shows that FAM83H-AS1 high expression indicated unfavorable prognosis in luminal breast cancer and was an independent prognostic indicator. To the best of our knowledge, this is the first report that suggests variable interaction between FAM83H-AS1 and IHC-detected PR and ER in the clinical outcome context. Furthermore, we demonstrate that ER and PR expression levels can act as potentiators of FAM83H-AS1 poor OS prediction. In particular, ER and PR high expression levels, together with FAM83H-AS1 over-expression, confers a very high risk (HR = 57) of decease in BRCA patients. This data suggest an important clinical role for FAM83H-AS1 in ER/PR positive breast cancer. It is currently unknown, however, if these statistical and clinical interactions are reflected at the biological or molecular level, and future studies must address this issue.

We were also able to find a significant correlation between FAM83H-AS1 high expression and poor tamoxifen response in BRCA patients. This association could partially explain the reduced clinical response in FAM83H-AS1 high expression group.

We also report that FAM83H-AS1 over-expression in TCGA breast cancer samples is associated with down-regulation of migration and cell death-related transcripts, like *FGF4, FGF21, LEP, CLDN17, TP53, BAX* and *TNFRSF11B.* Accordingly, we also found that FAM83H-AS1 knockdown significantly deregulates migration and apoptosis-related genes, such as *TP63* and *CLND1*. Transwell migration assays showed that indeed, cellular migration increases after FAM83H-AS1 silencing. LEP and CLDN1 had been both shown to induce cellular migration and epithelial to mesenchymal transition (EMT) in breast cancer cells^19–23^, further suggesting a FAM83H-AS1 role in the early steps of migration. Taken together, this data might explain the underlying mechanisms related to FAM83H-AS1 cell migration impairment in breast cancer cells.

FAM83H-AS1 might play a dual role, probably due to cellular context. FAM83H-AS1 was involved in regulation of cell proliferation, migration and invasion processes that were decreased after FAM83H-AS1 knockdown in lung cancer cells. Further analysis indicated the cell cycle was arrested at the G2 phase after FAM83H-AS1 knockdown^11^ In the same report, they found that MET/EGFR signaling was regulated by FAM83H-AS1. These conflicting results may be due to cellular context or specific regulation mechanisms, and henceforth, specific molecular targets.

We also identified that FAM83H-AS1 overexpression is associated with down-regulation of cellular death-related transcripts, like *BAX, TNFRSF11B* and *P53*. In vitro assays also show that FAM83H-AS1 silencing increases cellular death, possibly by up regulating genes like *p63.* One previous report^14^ showed that cell death was markedly increased after with FAM83H-AS1 knockdown in colorectal cell lines. FAM83H-AS1, Notch1 and Hes1 were significantly increased in colorectal cancer samples and cell lines. Cell proliferation was inhibited with FAM83H-AS1 knockdown and this effect mediated by FAM83H-AS1 could be reversed by Notch1 regulators^14^.

It is currently not clear, however, if FAM83H-AS1 has a direct or an indirect effect in gene regulation. In this regard, it has been shown that FAM83H‐AS1 epigenetically silenced *CDKN1A* by binding to EZH2 in glioma cells^24^. In our differential expression analysis, we demonstrate that FAM83H-AS1 is mostly down-regulating gene expression. This data might suggest an inhibitory regulation role for FAM83H-AS1. Future studies must address these mechanisms; as we cannot discard that FAM83H-AS1 may regulate master gene expression via recruiting epigenetic complexes (e.g. EZH2). In addition, the exact role for FAM83H-AS1 in up-regulated genes remains obscure. We cannot discard a subtle, alternative role for this lncRNA in gene activation, and future studies must address this issue.

Our results also show that FAM83H-AS1 is present both in nucleus and cytoplasm of breast cancer cells. It is possible that this lncRNA is playing a different role in cytoplasm, and future studies must focus on this question.

In conclusion, FAM83H-AS1 is a lncRNA that is de-regulated in multiple cancers, and is a promising molecule that can perform as an independent prognostic factor in ER/ PR positive breast cancer. FAM83H-AS1 deregulation is associated with migration and cell death impairment in BRCA samples and breast cancer cells, and may regulate a plethora of cancer-related gene targets, such as *p63, BAX, LEP* and *CLDN1.*

## Methods

### The Cancer Genome Atlas (TCGA) and Gene expression omnibus (GEO) datasets

FAM83H-AS1 expression levels were screened in the 33 tumor datasets (see supplementary Table 3 for details) from TCGA and correspondent normal tissues using the Gene expression Profiling Interactive Analysis (GPIA) platform (https://gepia.cancer-pku.cn/). The 33 tumors included are enlisted as follows: Acute myeloid leukemia (LAML); Adrenocortical carcinoma (ACC), Bladder Urothelial Carcinoma (BLCA); Brain Lower Grade Glioma (LGG); Breast invasive carcinoma (BRCA); Cervical squamous cell carcinoma and endocervical adenocarcinoma (CESC); Cholangiocarcinoma (CHOL); Chronic Myelogenous Leukemia (LMCL); Colon adenocarcinoma (COAD); Esophageal carcinoma (ESCA); Glioblastoma multiforme (GBM); Head and Neck squamous cell carcinoma (HNSC); Kidney Chromophobe (KICH); Kidney renal clear cell carcinoma (KIRC); Kidney renal papillary cell carcinoma (KIRP); Liver hepatocellular carcinoma (LIHC); Lung adenocarcinoma(LUAD);Lung squamous cell carcinoma (LUSC); Mesothelioma (MESO); Ovarian serous cystadenocarcinoma (OV); Pancreatic adenocarcinoma (PAAD); Prostate adenocarcinoma (PRAD); Rectum adenocarcinoma (READ); Sarcoma (SARC); Skin Cutaneous Melanoma (SKCM); Stomach adenocarcinoma (STAD); Testicular Germ Cell Tumors (TGCT); Thyroid carcinoma (THCA);Uterine Carcinosarcoma (UC);Uterine Corpus Endometrial Carcinoma (UCEC); Uveal Melanoma (UVM).

Potential target genes expression correlation, Hazard ratio (HR) map, co-expression map and BRCA stage plots were also generated in the GPIA platform. FAM83H-AS1 expression levels were considered significantly correlated with tumors when log2FoldChange > 1 and *p* value < 0.01.

Microarray generated expression data was downloaded from the GEO dataset GSE115577. This dataset includes RNA levels from 467 BRCA samples, analyzed with the Affymetrix HTA 2.0 platform. Downstream analysis is described below.

### IHC-detected hormonal receptors and FAM83H-AS1 risk model

Clinical information of the BRCA patients was downloaded from the TCGA database (https://portal.gdc.cancer.gov/). FAM83H-AS1 expression levels were downloaded from the TANRIC tool (https://ibl.mdanderson.org/tanric/_design/basic/main.html). We first searched for the ER and PR status, and the numerical value for percent stained cells, also available in the clinical data. We then calculated the Allred score^17^ (which measures the stain intensity and stain pattern) for ER and PR positivity levels in TCGA samples. We calculated the FAM83H-AS1 expression quartiles and stratified its levels of expression in this four groups (quartiles).

We then multiplied the Allred score with the FAM83H-AS1 expression levels (quartiles), and obtained nine patient groups: all the possible combinations for this particular model. We then performed two regression Cox models: in the first one, we established major risk groups. In the second one, we computed these major risk groups, shown in the results section.

### Breast cancer samples differential expression analysis

Breast cancer RNAseq counts were downloaded from the TCGA Data Portal (https://portal.gdc.cancer.gov). After dataset preparation, we identified the FAM83H-AS1 ID (ENSG00000282685) and downloaded the expression counts. Transcripts with 10 counts or less were not included in the analysis. In order to generate the high and low FAM83H-AS1 expression groups, we calculated two percentiles from the count expression data. The first quartile (25) contains the lowest FAM83H-AS1 expression counts, and the upper quartile (75), contains the highest expression levels for this transcript. We then performed differential expression analysis with the DESeq2 module from the Gene Pattern platform (https://software.broadinstitute.org/cancer/software/genepattern/). Genes were considered differentially expressed when Log2Fold Change was > 1.5 and − <1.5 and *p* adjusted value < 0.05. Volcano plots were generated with the Enhanced Volcano R Package.

The upper (75) and lower quartile (25) approach to generate FAM83H-AS1 expression groups described above was also performed with the expression data from GSE115577. In this particular case, groups were generated in order to perform single sample Gene Set Enrichment Analysis (ssGSEA).

### Breast cancer patients and biological samples

A total of 42 biological samples (biopsies) were collected from breast cancer patients attending to Fundación Cáncer de Mama (FUCAM) in Mexico City, Mexico. Adjacent normal samples were obtained from 2 cm above the surgical tumor margin. All patients signed a written informed consent before participating in this study. The study was approved by the Research Ethics Committee (INMEGEN) and the FUCAM Ethics Committee (Registration number: CE2009/11). All research methods were performed in accordance with relevant regulations and guidelines.

Biological samples were bisected; one portion was fixed in formaldehyde (10%), paraffin embedded (Paraplast Plus®; Sigma Aldrich ®, St Louis, Missouri, USA) and then submitted to haematoxylin and eosin staining for histopathological examination by an expert pathologist. Tumor stage was assessed, according to International standards. The second portion the sample was used for RNA extraction and functional downstream analysis. All tissues were liquid nitrogen-frozen and stored at − 80 °C.

In all 42 cases, demographic (age, sex), clinical (date of diagnosis, therapy received), pathologic (stage, grade, histological type) and prognostic data (recurrence, progression and overall survival) were available and correlated with FAM83H-AS1 expression status.

### Cell culture

ER/PR-positive human breast cancer cell line MCF-7 (ATCC HTB-22) cells were purchased from American Type Culture Collection (ATCC, Manassas, Virginia, USA) and cultured in Dulbecco´s Modified Eagle Medium F12 (DMEM-F12; Corning® Inc, N.Y, USA) with 10% Fetal Bovine Serum (Corning® Inc, N.Y, USA). Cells were grown in 75 cm^3^ cell culture bottles (Corning® Inc, N.Y, USA) at 37 °C with an atmosphere 95%/5% of air/CO_2_.

### Plasmids and transfection

Control and target plasmids were cloned using the BLOCK-iT™ U6 RNAi Entry Vector Kit (ThermoFisher™ Scientific, Waltham, Massachusetts, USA), according to the manufacturer´s recommendations. Short hairpin RNA oligos were designed using the Invitrogen Block-iT™ RNAi designer tool. Oligos were designed to target FAM83H-AS1 Exon 1 sequence (Oligo sh Top sequence: CACCGAAGAACATCCCAGATTACCCGCGAACGGGTAATCTGGGATGTTCTTTT; Bottom sequence: AAAAGAACATCCCAGATTACCCGTTCGCGGGTAATCTGGGATGTTCTTC). This double stranded oligo was then annealed and cloned into the entry vector pENTR™/U6 (ThermoFisher™ Scientific, Waltham, Massachusetts, USA). The plasmids were then introduced onto *E. coli* TOP10 competent cells, which were grown in LB/agar medium at 37 °C.

Plasmids were purified using GeneJet Plasmid Miniprep Kit (ThermoFisher™ Scientific, Waltham, Massachusetts, USA) and sequenced to confirm insert integrity.

Transfection experiments were performed using Xfect™ Transfection Reagent (Clontech Laboratories Inc., Mountain View, California, USA) following the manufacturer´s instructions. Briefly, 100,000 cells were cultured 24 h prior to transfection in 24-well plates. 750 ng of the random plasmid and 750 ng of sh-FAM83H-AS1 plasmid were diluted and then added to each well. Transfection reaction was incubated for 24 h; medium was removed and replaced with fresh complete medium.

### RNA extraction

Genomic RNA was extracted using the commercial kit AllPrep® DNA/RNA FFPE (Qiagen® Inc, Valencia, CA) following manufacturer´s instructions. Briefly, the tissues were deparaffinized, disrupted and lysed. RNA was then precipitated, washed, purified and suspended in RNAse free water. RNA concentration was evaluated by spectrofotometry (NanoDrop Technologies, Wilmington, Delaware, USA). RNA integrity was analized using the BioAnalyzer 2100 (Agilent Technologies, Palo Alto, CA, USA). Samples were stored at − 80 °C.

### *Quantitative reverse transcription polymerase chain reaction (qRT-PCR*)

cDNA was synthetized using SuperScript III RT-PCR (Invitrogen, ThermoFisher™ Scientific, Waltham, Massachusetts, USA) following the manufacturer’s instructions. Briefly, 100 ng of total RNA from cell lines or breast cancer samples were used to synthesize cDNA in a final reaction volume of 20 μL. The PCR reaction contained 1 μL of cDNA, 5 μL 2X TaqMan Universal Master Mix (Applied Biosystems, ThermoFisher™ Scientific, Waltham, Massachusetts, USA), 0.5 μL TaqMan probes (AIS09YL custom for FAM83H-AS1) and 3.5 μL of nuclease-free water. Both primers and reporter were designed to target FAM83H-AS1 exon 1 (Forward primer: ATCCCAGTTGATATCAGGGCAATC; reverse primer: TGTAAGCCCTTGATATTGG; reporter: TCCTGGCTGTTTTCC). GAPDH (Hs99999905) and SCARNA5 (Hs03391742_cn) transcripts were used as endogenous controls.

### Subcellular fractionation and validation

MCF7 cellular fractionation (cytoplasm and nucleus) assays were performed using the Protein and RNA Isolation System (PARIS™) Kit (ThermoFisher™ Scientific, Waltham, MA, USA) according to manufacturer’s instructions. Total and fractionated RNA was purified and then analyzed by RT-PCR, as described above. RNA percentages of each transcript over total RNA were calculated. GAPDH (Hs99999905) and MALAT-1 were used as cytoplasmic and nuclear controls, respectively.

The lncATLAS database (https://lncatlas.crg.eu/) was used to validate FAM83H-AS1 subcellular localization in MCF7 cells.

### Microarray expression analysis

Global expression analysis was performed using Human Transcriptome Array 2.0® (Affymetrix® Inc, Santa Clara, CA, USA). This array covers 44,699 coding RNAs and 22,829 non-coding RNAs. A total of 200 nanograms of RNA were processed in each assay. All samples were processed using WT Plus Reagent Kit and Affymetrix hybridization kits, according to Affymetrix® recommendations.

### Gene expression profiles

Affymetrix HTA 2.0 dataset analysis was performed using the Affymetrix® Expression Console and Transcriptome Analysis Console®. Normalized intensities from the sh-RANDOM condition was compared to normalized intensities from the sh-FAM83H-AS1 condition using one-way ANOVA. Genes were considered differentially expressed when fold change was > 1.5 and −  <1.5 and *p* value < 0.05.

### Pathway enrichment analysis

Pathway enrichment analysis was performed with Ingenuity Pathway Analysis® (IPA) software. Z-scores and *p* values were also computed using this platform. Only differential statistically significant genes were included in this analysis (see criteria above).

Gene set enrichment analysis (GSEA) was performed with the Web-based Gene SeT Analysis Toolkit (WebGestalt) platform (www.webgestalt.org). Non-significant pathway-enriched genes were included in this analysis as a priori set of genes.

ssGSEA was performed with the ssGSEA projection Gene Pattern module (https://software.broadinstitute.org/cancer/software/genepattern/). Graphic visualization was constructed using the Constellation Map module^25^, also available in the Gene Pattern platform. Migration and cell death complete pathways that were generated in the IPA software, as a result of the microarray expression assays, were used as a priori set of genes. GSE115577 dataset (BRCA samples) was used as the input cohort to perform enrichment analysis.

### Transwell migration and invasion assays

For migration assays, MCF-7 cells were seeded in 24-well plates (100,000 cells per well) and transfected with sh-RANDOM or sh-FAM83H-AS1 plasmids. 24 h after transfection, cells were tripsinized and cultured in Transwell® Chambers (8.0 µm) (Corning® Inc, N.Y, USA). Cells were incubated for 24 and 48 h after tripsinization and then fixed with cold 70% Ethanol (J.T. Baker®, Fisher Scientific, Waltham, Massachusetts, USA). Cells were stained with 1X SRB Staining Solution (VitroSure™ SRB Viability/Cytotoxicity Assay Kit, GeneCopoeia™, Rockville, Maryland, USA). In vitro invasion assays were done in the same fashion, but adding Matrigel Matrix (Corning® Inc, NY, USA) in each Transwell® Chamber. Invasion was analyzed after 24 and 48 h after seeding.

Cell migration and invasion was evaluated by double-blind manual counting and image analysis using the software ImageJ.

### Caspase 3 activity assays

Cell death induction was evaluated using the Caspase-3 Colorimetric Activity Assay Kit (Merck Millipore®, Burlington MA, USA), following the manufacturer´s instructions. Briefly, MCF7 cells were transfected with sh-RANDOM, sh-FAM83H-AS1 or treated with dimethyl sulfoxide (DMSO) 10%. Caspase 3 activity was measured 24 h, 48 h and 72 h after transfection or DMSO treatment.

### Statistical analysis

Kaplan Meier survival analysis for FAM83H-AS1 associated tumors (except for BRCA) were performed in the GPIA platform.

Overall survival (OS) of the BRCA TCGA patients and our independent cohort was analyzed with the Kaplan–Meier model and the multivariable Cox´s regression model. This analysis was performed with the PASW statistics software (SPSS, IBM®, Quarry Bay, Hong Kong). Fischer´s exact test was calculated in order to correlate clinical variables with FAM83H-AS1 expression level. Student´s T-tests were performed to calculate statistical differences in functional in vitro assays. For all statistical tests, the level of significance was < 0.05.

### Ethics approval and consent to participate

This study was approved by the Research and Ethics Committee of National Institute of Genomic Medicine and the Institute of Breast Diseases, FUCAM (Registration number: CE2009/11). Written informed consent was obtained from each patient before any procedure.

## Supplementary information

Supplementary information.

## Data Availability

Gene Expression Omnibus (GEO): data submitted.
