# Supplementary material for: FAM83H-AS1 is a potential modulator of cancer driver genes across different tumors and a prognostic marker for ER/PR + BRCA patients
Source: Sci Rep. 2020 Aug 24;10:14145. doi: 10.1038/s41598-020-71062-2 (PMC7445275; doi:10.1038/s41598-020-71062-2)

FAM83H-AS1 is a potential modulator of cancer driver genes across different tumors and a prognostic marker for ER/PR+ BRCA patients

Ríos-Romero Magdalena, Cedro-Tanda Alberto, Peña-Luna Monica, Mancera-Rodríguez Marco, Hidalgo-Pérez Lizbett[1], Cisneros-Villanueva Mireya, Beltrán-Anaya Fredy Omar, Arellano-Llamas Rocío, Jiménez-Morales Silvia, Alfaro-Ruiz Luis Alberto, Tenorio-Torres Alberto, Domínguez-Reyes Carlos, Villegas-Carlos Felipe, Ochoa-Mendoza Elsa, Hidalgo-Miranda Alfredo.

Ríos-Romero M, *et al.,* 2020. FAM83H-AS1 is a potential modulator of cancer driver genes across different tumors and a prognostic marker for positive ER/PR receptor breast cancer patients

Supplementary table 1. Cox regression analysis for PR positive breast cancer TCGA cohort

	95.0% CI for Exp(B)							
	B	SE	Wald	df	Sig.	Exp(B)	Lower	Upper
FAM83H-AS1 expression	0.435	0.22	3.93	1	0.047	1.545	1.005	2.376
Other			6.699	2	0.035			
PR (Positive)	0.432	0.221	3.832	1	0.05	1.541	0.999	2.375
PR (Negative)	0.981	0.477	4.23	1	0.04	2.666	1.047	6.787

Supplementary table 2. Cox regression analysis for ER positive breast cancer TCGA cohort

	95.0% CI for Exp(B)							
	B	SE	Wald	df	Sig.	Exp(B)	Lower	Upper
FAM83HAS1 median expression	0.411	0.218	3.569	1	0.059	1.508	0.985	2.31
Other			9.649	2	0.008			
ER (Positive)	0.299	0.239	1.571	1	0.21	1.349	0.845	2.154
ER (Negative)	1.305	0.433	9.089	1	0.003	3.689	1.579	8.618

Ríos-Romero M, *et al*., 2020. FAM83H-AS1 is a potential modulator of cancer driver genes across different tumors and a prognostic marker for positive ER/PR receptor breast cancer patients

Supplementary table 3. Tumor and normal sample sizes used in the TCGA analyses

**Cancer type**	**Sample size**	
	T	N
**ACC**	77	128
**BLCA**	404	28
**BRCA**	1085	291
**CESC**	306	13
**CHOL**	36	9
**COAD**	275	349
**DLBC**	47	337
**ESCA**	181	286
**GBM**	163	207
**HNSC**	519	44
**KICH**	66	53
**KIRC**	523	100
**KIRP**	286	60
**LAML**	173	70
**LGG**	518	207
**LIHC**	369	160
**LUAD**	483	347
**LUSC**	486	338
**MESO**	87	NA
**OV**	426	88
**PAAD**	179	171
**PCPG**	182	3
**PRAD**	492	152
**READ**	92	318
**SARC**	262	2
**SKCM**	461	558
**STAD**	408	211
**TGCT**	137	165
**THCA**	512	337
**THYM**	118	339
**UCEC**	174	91
**UCS**	57	78
**UVM**	79	NA

Ríos-Romero M, *et al.,* 2020. FAM83H-AS1 is a potential modulator of cancer driver genes across different tumors and a prognostic marker for positive ER/PR receptor breast cancer patients

Supplementary figure 1. Survival curve for TCGA BRCA PR negative patients

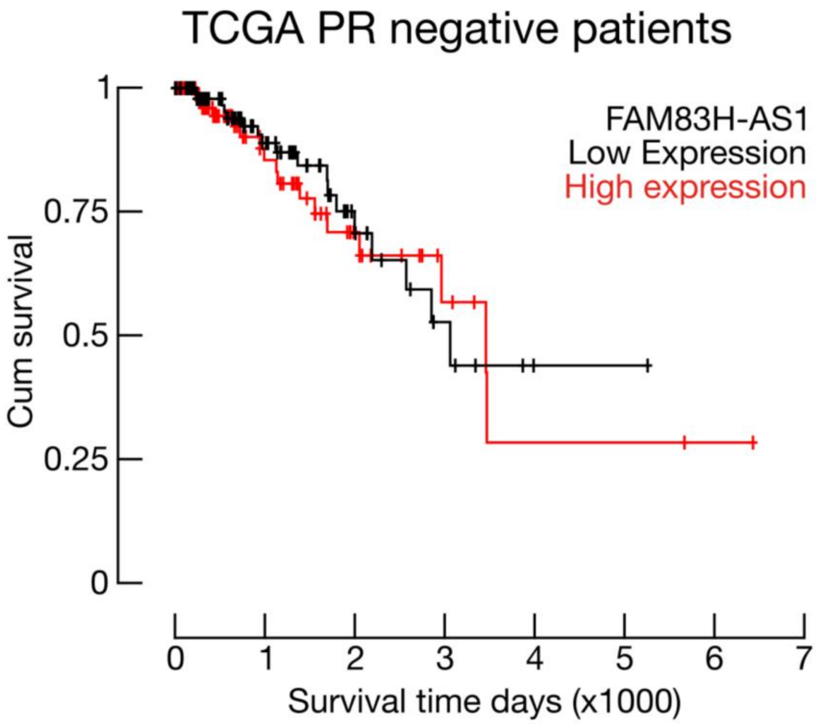

Ríos-Romero M, *et al.,* 2020. FAM83H-AS1 is a potential modulator of cancer driver genes across different tumors and a prognostic marker for positive ER/PR receptor breast cancer patients

Supplementary figure 2. Survival curve for TCGA BRCA ER negative patients

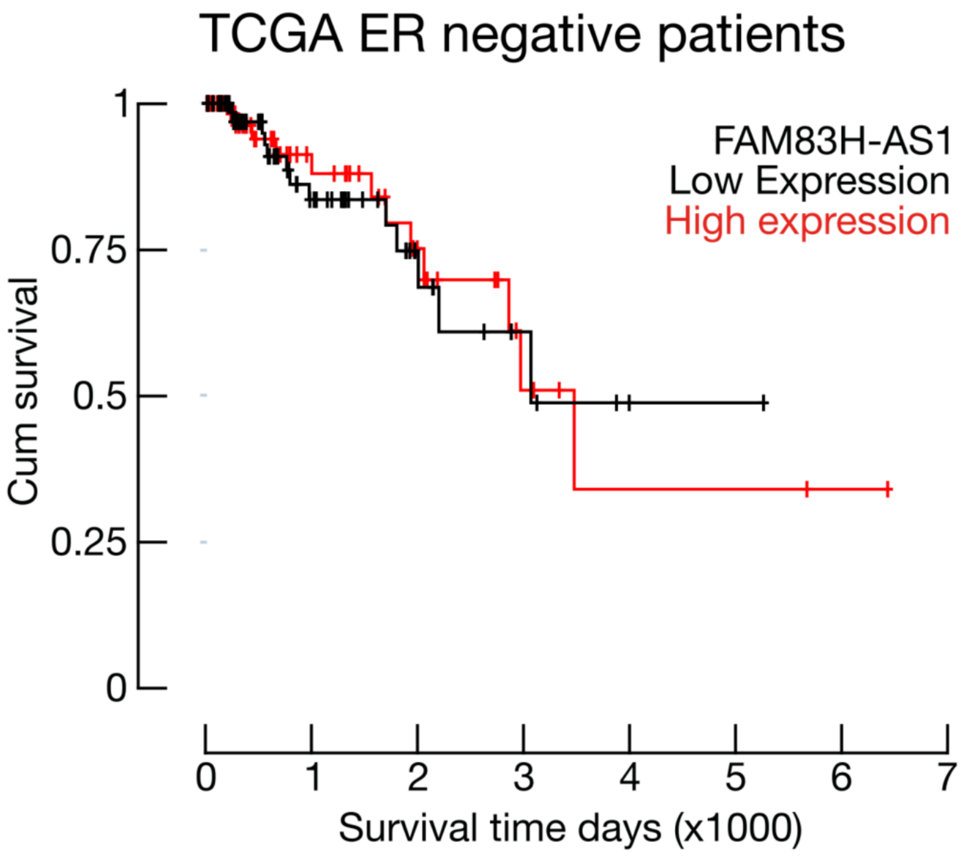

Ríos-Romero M, *et al.,* 2020. FAM83H-AS1 is a potential modulator of cancer driver genes across different tumors and a prognostic marker for positive ER/PR receptor breast cancer patients

Supplementary figure 3. Survival curve for TCGA SKCM patients

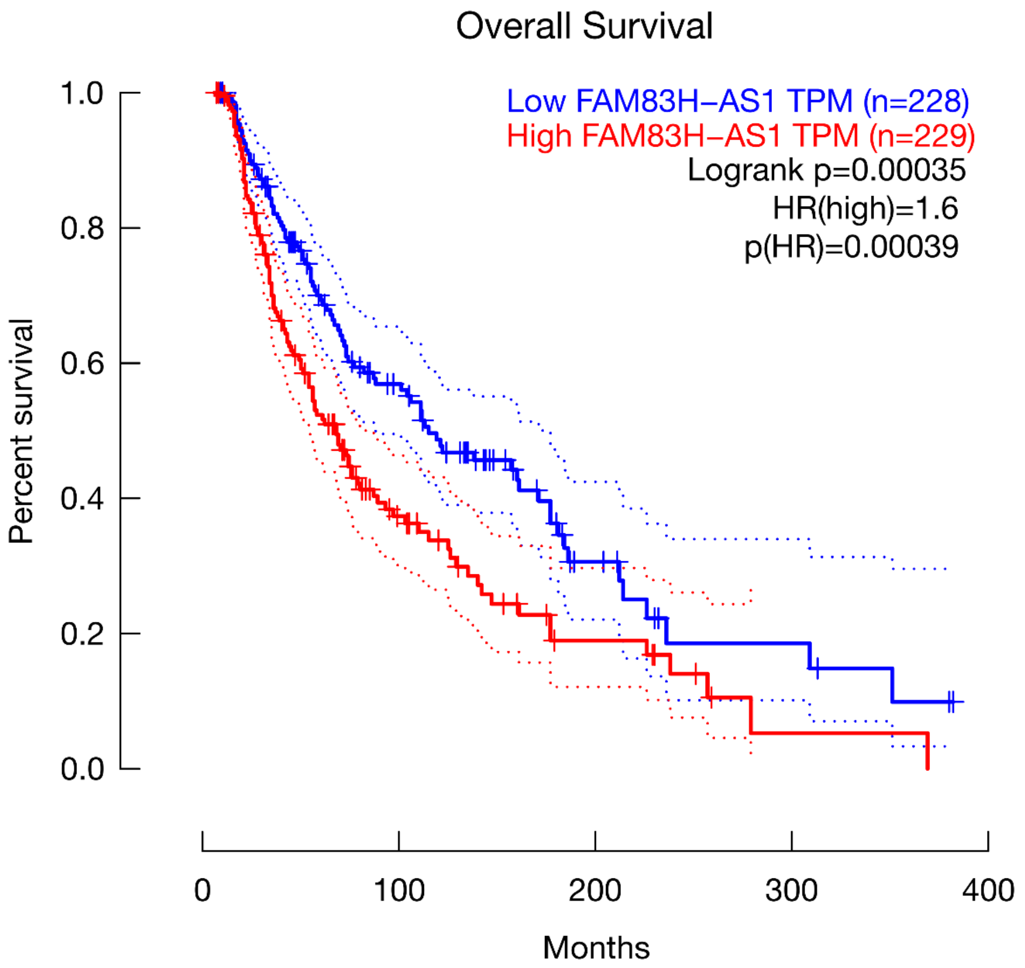

Ríos-Romero M, *et al.,* 2020. FAM83H-AS1 is a potential modulator of cancer driver genes across different tumors and a prognostic marker for positive ER/PR receptor breast cancer patients

Supplementary figure 4. FAM83H-AS1 is located both in the nucleus and cytoplasm in MCF7 cells

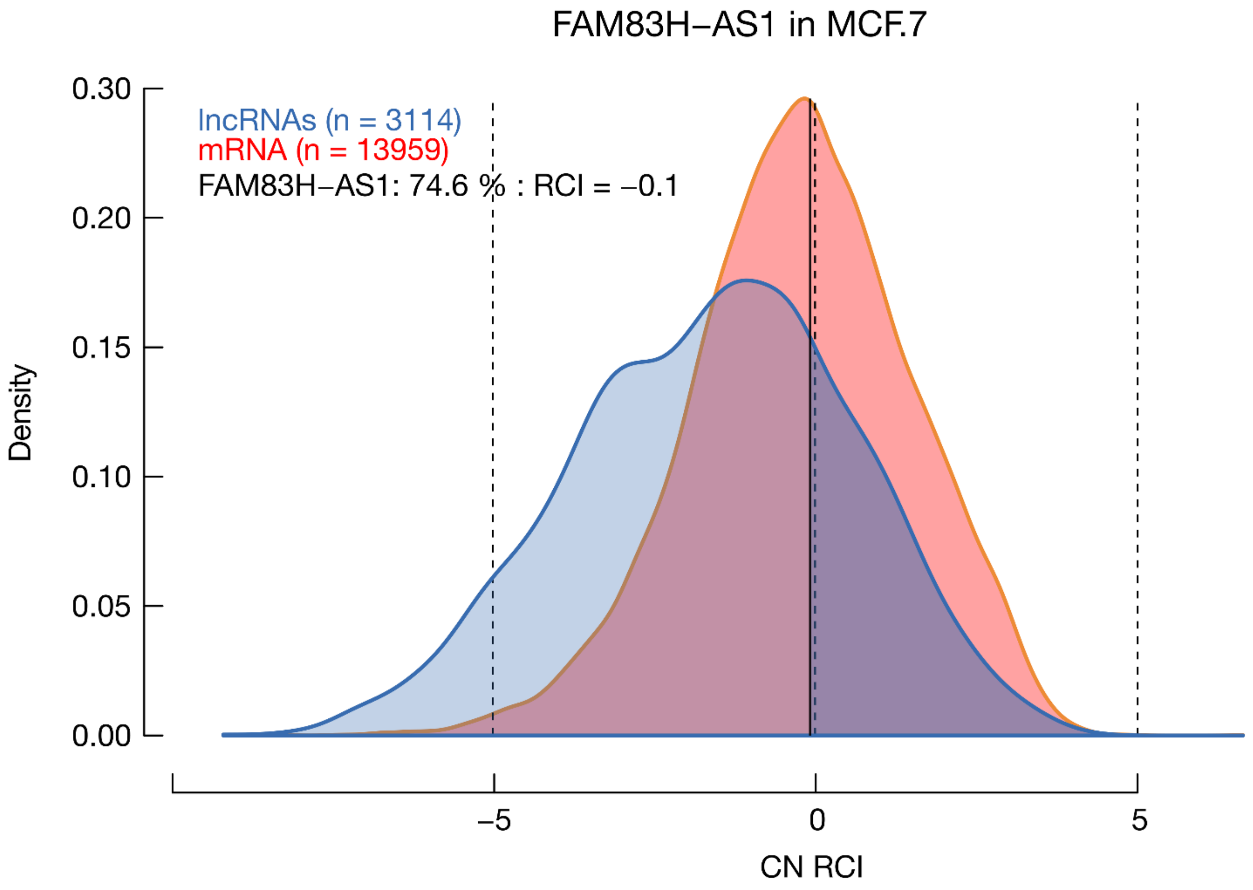

Ríos-Romero M, *et al.*, 2020. FAM83H-AS1 is a potential modulator of cancer driver genes across different tumors and a prognostic marker for positive ER/PR receptor breast cancer patients

Supplementary figure 4. FAM83H-AS1 is not involved in invasion in ER/PR positive MCF7 cells

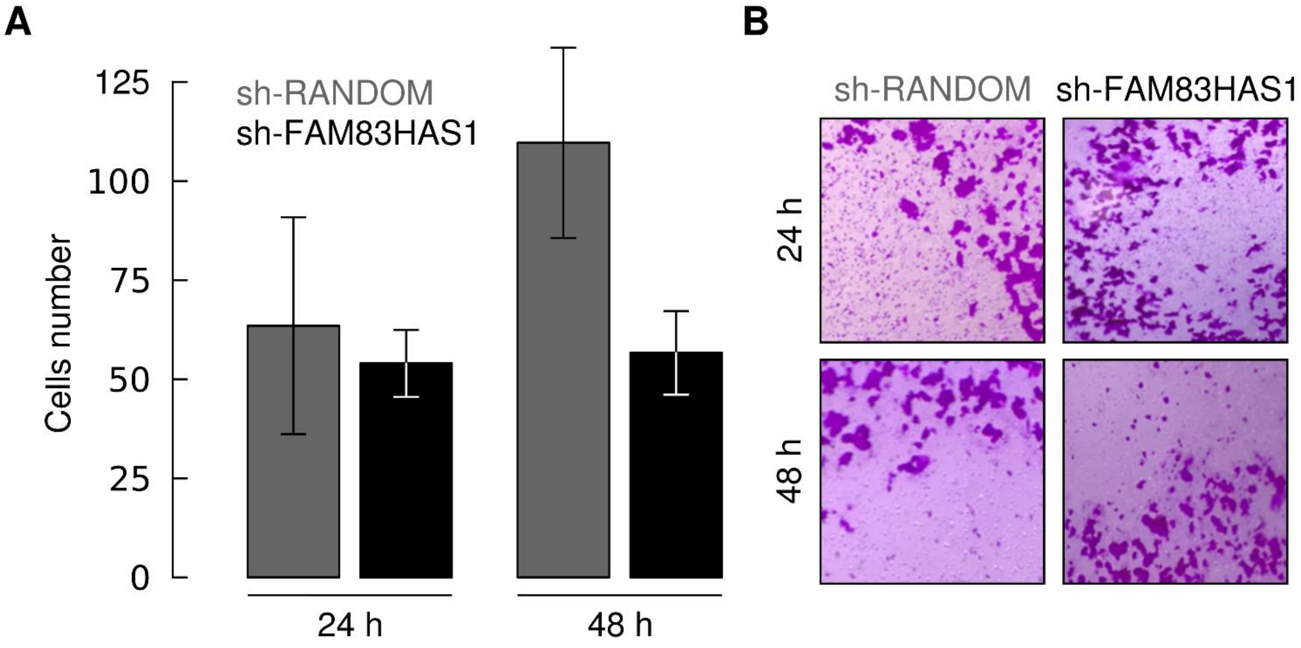

Supplement: Supplementary file 1 — Supplementary information. [file 41598_2020_71062_MOESM1_ESM.pdf]
